# Analysis of the clinical efficacy of “dynamic hook technique” combined with the Allgower-Donati suture method in the surgical incision for calcaneal fractures

**DOI:** 10.3389/fsurg.2025.1676761

**Published:** 2025-11-14

**Authors:** Bo Liu, Long Ma, Dawei Xiao, Cheng Chen, Hui Zeng, Honghai Wang, Bowen Zhang, Gangqiang Wu, Haiyang Yu

**Affiliations:** 1 Department of Orthopedics, Affiliated Fuyang People’s Hospital of Anhui Medical University, Fuyang, Anhui, China; 2 Department of Orthopedics, NO. 2 People’s Hospital of Fuyang City, Fuyang, Anhui, China; 3 Department of Orthopedics, Cancer Hospital of Fuyang City, Fuyang, Anhui, China

**Keywords:** calcaneal fracture, hook, dynamic hook technique, Allgöwer-Donati suture, complications, postoperative pain

## Abstract

**Objectives:**

To analyze the clinical outcomes of “dynamic hook technique” combined with the Allgöwer-Donati suture technique in reducing incisional complications and Incisional pain after calcaneal fracture surgery.

**Methods:**

Totally, 93 patients with calcaneal fractures who underwent open reduction and internal fixation were included and divided into control and observation groups. In the observation group, the flap was elevated using the “dynamic hook technique”, while in the control group, the flap was elevated using the static Kirschner needle lifting technique. The flap in the two groups were sutured using the Allgöwer-Donati sutures. Postoperative complications, including tension blisters surrounding the incision, necrosis of the incision flap, and the steel plate exposure were analyzed. The Visual Analog Scale (VAS) was utilized to assess postoperative incision pain at 3 days, 1 and 2 weeks post-surgery. All patients were followed up for a duration of 3–6 months, with suture removal occurring 2–3 weeks postoperatively.

**Results:**

The incidence of complications was significantly lower in the observation group than the the control group (4.5% vs. 18.4%, *P* < 0.05). The patients in the observation group had significantly lower VAS pain scores at each time point after surgery when compared to the control group (*P* < 0.05).

**Conclusion:**

The combination of the “dynamic hook technique” and the Allgöwer-Donati suture is effective in preventing incisional complications and alleviating postoperative pain after open reduction and internal fixation for calcaneal fractures.

## 1 Introduction

Calcaneal fractures are the most common tarsal fractures, with traumatic axial loading, such as heel strike when falling from a height as the common causes (1). Sanders type II–IV calcaneal fractures often require surgical treatment. Sanders III and Sanders IV calcaneal fractures are generally severely comminuted. Currently, the most classic surgical method is the use of the extensile lateral approach (ELA) to open a skin flap combined with internal fixation with a steel plate (2). Since the soft tissue around the calcaneus has less coverage and poor blood supply, early use of the ELA to lift the skin flap combined with steel plate internal fixation for the treatment of calcaneal fractures can lead to complications, such as necrosis of the broken end of the skin flap, infection, delayed healing, and steel plate (3–5). When using the ELA to fully expose the fracture end during calcaneal fracture surgery, a common method is to use three Kirschner wires to retract the skin flap. This method often increases the pressure on the skin flap and affects the skin if the operation time is long. The postoperative suture method is also very important. A previous study reported that compared with the improved interrupted vertical mattress suture (Allgöwer-Donati suture), the previous interrupted vertical mattress suture (Donati suture) significantly increased the blood supply to the incision flap, thus reducing the healing rate of the skin flap (6). In order to solve the above problems, this study used retractors to dynamically traction the skin flap (referred to as the “dynamic hook technique”), and adopted the modified interrupted vertical mattress suture method (Allgöwer-Donati suture method) to expose and suture the skin flap. The purpose of the study was to investigate the effect of dynamic hook technique combined with Allgöwer-Donati suture method in preventing incisional complications and pain after surgical treatment of calcaneal fractures.

## 2 Materials and methods

### 2.1 Participants

A total of 93 patients with calcaneal fractures who underwent open reduction and internal fixation through the ELA in NO.2 People's Hospital of Fuyang City and Fuyang Cancer Hospital from January 2019 to December 2023 were included. All patients were randomly grouped using a digital table method. There were 44 patients in the observation group and 49 patients in the control group. Patients in the observation group used the dynamic hook technique to open the skin flap during the operation, with Allgöwer-Donati suture method being used to suture the skin flap postoperatively. And Patients in the control group used the static K-wire lifting technique to open the skin flap during surgery, with the Allgöwer-Donati suture method being used to suture the skin flap postoperatively.

All patients had closed and simple calcaneal fractures that were classified using the Sanders classification (7). Patients who had other diseases, such as deep vein thrombosis of the lower limbs, diabetes, immune system diseases, tumors were excluded.

This study was approved by the Ethics Committee of NO. 2 People's Hospital of Fuyang City. All enrolled patients signed informed consent.

### 2.2 Preparation of soft tissue conditions at the incision site

All patients undergo swelling reduction of the affected foot prior to surgery, which is conducted when the skin wrinkle test yields a positive result. The skin wrinkle test is performed by placing the foot in slight dorsiflexion and eversion, followed by gentle compression of the skin above the lateral aspect of the calcaneus. A positive result is indicated by the presence of skin wrinkling.

### 2.3 Surgical technique

All patients were positioned in the lateral decubitus position during the procedure. An ELA was made on the lateral calcaneus, and the skin flap was meticulously dissected subperiosteally, with careful attention paid to protect the sural nerve throughout the operation.

In the control group, two commonly used Kirschner wires were secured at the anterior and posterior aspects of the talus during the process of opening the skin flap to expose the articular surface and facilitate reduction. Additionally, one Kirschner wire was fixed to the cuboid bone, and the bent Kirschner wire was utilized to assist in opening the flap.

In the observation group, two thyroid hooks were employed to elevate the skin flap, and these retractors were also used to maintain exposure of the joint surface during reduction. When opening the skin flap, the thyroid hook should be positioned perpendicular to the incision direction, ensuring full contact with the skin flap. During reduction, the thyroid hook's position must be adjusted according to the fracture block's location. After satisfactory reduction of the fracture ends, the thyroid hook should be released during fluoroscopy. While fixing the steel plate, the thyroid hook should be used to lift the flap corresponding to the screw during insertion. In this surgery, the thyroid hook not only provides a large contact area with the skin flap but also remains in a dynamic state; during intraoperative fluoroscopy, the skin flap is completely relaxed. These operational techniques facilitate blood reperfusion of the skin flap after traction. The principle of force control when lifting the skin flap during thyroid hook surgery is to fully expose the surgical field while performing gentle maneuvers. This approach is referred to as the “dynamic hook technique” proposed in this study.

The fractures in both groups were reduced and stabilized using calcaneal locking plates. The skin flaps in both the two groups were sutured using a modified interrupted vertical mattress suture technique, known as the Allgöwer-Donati suture method. This suturing technique involved the following steps: a Type 0 antibacterial absorbable suture was used, with the needle inserted from a distance away from the skin edge of the incision. The needle was then passed horizontally beneath the skin and emerged from the subcutaneous layer on the opposite side of the incision. The needle was turned to exit near the contralateral skin edge, thereby completing one suture. The suture was tied at the distal end of the incision, with an approximate distance of 1 cm maintained between each suture. Postoperatively, a negative pressure drainage tube was placed in all patients, and the incision was disinfected and bandaged.

### 2.4 Postoperative care

All patients received intravenous infusion of ibuprofen for 3 days after surgery. All patients elevated the affected limb to minimize swelling for 48 h post-surgery and administered antibiotics for 24 h post-surgery. The drainage tube was removed when the drainage volume fell below 20 ml within 12 h after surgery. The dressing was changed twice daily for the first three days following surgery, during which the blood supply to the skin flap was monitored. Subsequently, the dressing was changed every other day, and the sutures were removed between 14 and 21 days after surgery. All patients engaged in non-weight-bearing functional exercises for a duration of 12 weeks, after which they transitioned to weight-bearing functional exercises.

### 2.5 Postoperative observation indicators

Postoperative observation indicators included complications at the incision site, including epidermal necrosis, skin flap necrosis, and exposure of the steel plate. The visual analogue score (VAS) for surgical incision pain was assessed at 3 days, 1 and 2 weeks post-surgery.The VAS scores for the surgical incisions were all completed by the patients.

### 2.6 Statistical analysis

Statistical analysis was conducted using SPSS version 21.0. The normality of continuous variables was evaluated with the Shapiro–Wilk test. Quantitative data that followed a normal distribution were expressed as mean ± standard deviation and analyzed using the *t*-test. In contrast, quantitative data that exhibited a skewed distribution were reported as median (first quartile, third quartile) [M (Q1, Q3)] and analyzed using the Mann–Whitney *U* test. Categorical data were presented as [cases (%)] and analyzed using either the *χ*^2^ test or Fisher's exact test. A *P* value of less than 0.05 was deemed statistically significant.

## 3 Results

**Figure 1:**
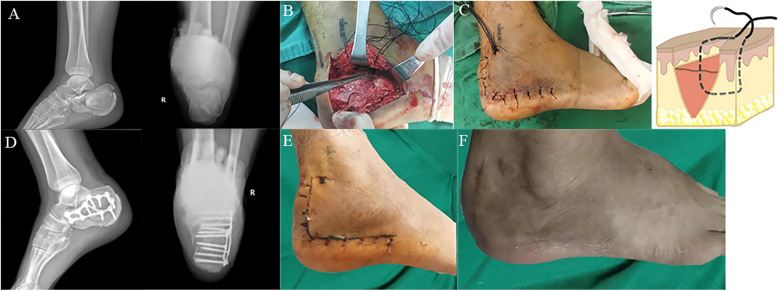
**(A)** Lateral and harris view radiographs of the right displaced intra-articular calcaneus fracture with significant articular depression and comminution; **(B)** the “dynamic hook technique” exposes the flap; **(C)** Allgöwer-Donati suture flap and suture schematic diagram; **(D)** lateral and harris view radiographs of the right calcaneus status post fixation using fragment plate; **(E)** the incision was sutured 2 weeks after operation, and class A healing was achieved; **(F)** follow up of incision conditions at 3 months after surgery.

No statistically significant differences were observed in age, gender, fracture type, injury factors, or smoking status between the two groups (Table 1). All patients were followed up for a duration of 3–6 months, with suture removal occurring 2–3 weeks postoperatively. The waiting time for surgery, operation time, incision suturing time, and drainage tube removal time did not differ significantly between the two groups (Table 2). Typical cases in the observation group are shown in Figure 1.

**Table 1:** Comparison of general clinical data between two groups of patients.

Factors	Observation group	Control group	*P*-value
Sex			0.409
Man	41	42	
Woman	3	7	
Mean age (years)	47.3 ± 10.7	47.8 ± 10.3	0.813
Sanders type			0.850
II	14	18	
III	22	3	
IV	8	21	
Injury type			0.713
Falling	18	20	
Car accident	3	6	
Falling injury	21	23	
Smoke	10	15	0.392

**Table 2:** Comparison of the perioperative general condition and incisional complications between the two groups (mean ± SD).

Factors	Observation group	Control group	*P*-value
Waiting time for surgery (days)	10.20 ± 1.69	10.61 ± 1.73	0.255
Surgical time (minutes)	81.1 ± 13.80	83.1 ± 14.80	0.498
Suture time of incision (minutes)	12.34 ± 1.14	12.49 ± 1.19	0.541
Removal time of incision drainage tube (days)	2.64 ± 0.49	2.59 ± 0.50	0.664
Complications [cases (%)]	2 (4.5%)	9 (18.4%)	0.039
Epidermal necrosis	2	6	
Flap necrosis		2	
Steel plate exposure		1	

In terms of incisional complications after surgery, in the observation group, epidermal necrosis was found in 2 patients, skin flap necrosis and steel plate exposure were not noted, the overall complication incidence was 4.5%. Conversely, in the control group, 6 patients experienced epidermal necrosis, 2 patients developed skin flap necrosis, and 1 patient developed steel plate exposure, the complication incidence rate was 18.4%. There were statistically significant differences in the complication incidence rate between the two groups (*P* < 0.05, Table 2).

**Figure 2:**
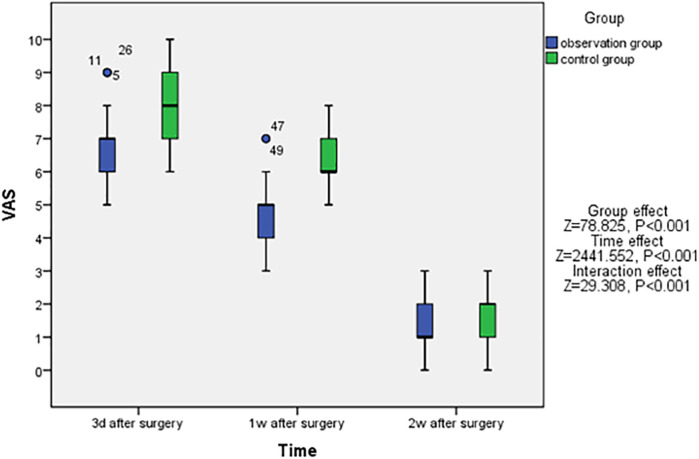
Change trends of postoperative VAS scores in two groups.

Furthermore, a reduction in the VAS scores was observed in both the observation and control groups. And patients in the observation group had significantly lower VAS pain scores at each time point after surgery when compared to the control group (*P* < 0.05, Figure 2).

## 4 Discussion

Calcaneal fractures commonly result from falls from a height that impact the heel. The calcaneus has a limited blood supply, minimal soft tissue coverage, and a complex anatomical structure. Recent studies have highlighted that minimally invasive surgery for calcaneal fractures can significantly decrease postoperative complications; however, some experienced surgeons still prefer the ELA as the surgical approach (8, 9). The primary issues associated with the ELA in calcaneal fracture surgery are its extensive scope and the trauma it inflicts. Postoperative skin flap necrosis at the incision site can easily occur, potentially leading to deep infections and other complications that may significantly impact patients' quality of life (3). Recent research involving freshly frozen cadavers suggests that positioning the vertical edge of the incision along the lateral edge of the Achilles tendon during the lateral calcaneal approach may help avoid damage to the calcaneal branch of the peroneal artery, which supplies the lateral calcaneal flap (10). This adjustment could reduce the incidence of incisional necrosis; however, clinical trials to support this finding are currently lacking (10). Consequently, it is crucial to carefully consider preoperative timing, intraoperative techniques, and postoperative wound monitoring when undertaking surgical treatment for calcaneal fractures.

### 4.1 Preoperative factors

When using the ELA for calcaneal fractures, it is crucial to consider the timing of surgery, as this can significantly reduce complications associated with surgical incisions (11). Following a calcaneal fracture, the soft tissue surrounding the foot experiences considerable swelling. In the case of the ELA, it is generally necessary for the swelling to subside almost completely, which typically requires an extended period (12). All patients received soft tissue ice packs, had their affected limbs elevated, and engaged in various foot activities to mitigate swelling prior to surgery. Surgical intervention was deemed possible only after wrinkles appeared in the surrounding soft tissue skin. In this study, both the observation group and the control group underwent surgical treatment after the appearance of wrinkles in the calcaneal soft tissue, thereby eliminating this factor as a potential confounder. Risk factors for incisional complications following closed calcaneal fractures include surgery performed within 7 days of the fractures, surgical durations exceeding 1.5 h, absence of postoperative drainage, static skin dispersion, and patient smoking (13). Recent research indicates that smoking has the most significant adverse effect on the healing of skin flaps after calcaneal fracture surgery (14). Consequently, it is imperative for all patients preparing for surgery to abstain from smoking both prior to the procedure and until the wound has fully healed. In this study, 2 patients in the observation group who experienced epidermal necrosis had a long-term smoking history prior to surgery, while in the control group, five out of eight patients with postoperative incisional complications also had a long-term smoking history. However, there was no statistically significant difference in the comparison of postoperative incision complications between these two groups concerning smoking factors (*P* > 0.05), thereby excluding the influence of this variable on the findings of this study.

### 4.2 Intraoperative factors

The duration of the operation and the protection of the skin flap are critically important during surgical procedures. Prolonged operation times significantly increase the postoperative incision infection rates (15), while the use of a tourniquet can notably reduce the duration of surgery (16). In this study, tourniquets were employed on all patients. The operation times for the two patient groups did not significantly impact the study outcomes (*P* > 0.05, Table 2). Experienced surgeons can substantially lower the infection rates of incisions, achieving rates as low as 1.8% (17, 18). This study was conducted by two senior physicians. Previously, three Kirschner wires were utilized to elevate the skin flap during fracture reduction. Extended operation times resulted in prolonged pressure from the Kirschner wires on the skin flap, adversely affecting its blood supply. To address these concerns, we adopted the use of two thyroid retractors to elevate the skin flap during surgery. The larger contact area between the thyroid retractors and the skin flap allows for better control of the pressure exerted during the procedure. Furthermore, if the operation time extends or repeated fluoroscopy is necessary, the thyroid retractors can be loosened to restore ischemic perfusion to the skin flap, thereby reducing the incidence of postoperative complications. This approach is referred to as “dynamic hook technique”. In this study, full-thickness incisions were performed on anesthetized pigs, employing various wound closure techniques, including simple, vertical mattress, horizontal mattress, and Allgöwer-Donati suture. It was determined that the Allgower-Donati suture method had the least detrimental effect on cutaneous blood flow at the wound margins (19). Additionally, the use of a finger retractor in conjunction with a modified interrupted vertical mattress suture (Allgöwer-Donati suture) significantly reduced flap necrosis postoperatively (20). The choice of suturing technique is crucial in minimizing complications associated with incisions for calcaneal fractures. Specifically, the modified interrupted vertical mattress suture (Allgöwer-Donati suture) has been shown to markedly decrease postoperative complications and enhance incision healing. In this study, all flaps in the middle and late stages of the 43 patients treated for calcaneal fractures using the “dynamic hook technique” alongside the Allgöwer-Donati suture method achieved complete healing, with only two cases of incisional epidermal necrosis reported. Notably, the Allgöwer-Donati suture method was also utilized in the control group, demonstrating its effectiveness in both the observation and control groups. Furthermore, the intraoperative “dynamic hook technique” exhibited a more pronounced impact on the study of postoperative incision complications.

### 4.3 Postoperative factors

All patients had negative pressure drainage tubes placed postoperatively. The use of a negative pressure drainage tube following surgery can alleviate pressure on the skin flap resulting from bleeding at the calcaneal fracture site, thereby facilitating incision healing. The drainage tube remains in place for 1–3 days post-surgery and is removed when the drainage volume within a 24 h period falls below 20 ml.

In this clinical study, the author posits that utilizing the ELA to treat calcaneal fractures necessitates careful protection of the skin flap during the procedure to minimize incision-related complications. Previously, the author opened the skin flap and employed three Kirschner wires along with a modified interrupted vertical mattress suture (Allgöwer-Donati suture) for closure. Currently, the author utilizes two thyroid retractors to dynamically elevate the skin flap in conjunction with the modified interrupted vertical mattress technique. The incision is subsequently sutured using the Allgöwer-Donati method. After controlling for other variables that may influence the incision flap, it is evident that the application of “dynamic hook technique” during surgery not only significantly reduces postoperative incision complications (as shown in Table 2) but also alleviates postoperative incision pain (Figure 2).

This study presents several limitations. First, the study does not investigate whether this technology can mitigate clinical complications and incision pain under conditions that may affect postoperative incision healing. Second, the sample size of this study is relatively small, further studies with larger sample size are required. Furthermore, the research was conducted at two local tertiary hospitals, where the intensity of assistance using the thyroid retractor varied. Future studies should involve the same surgeon performing surgical operations with consistent assistants to minimize iatrogenic interference. Additionally, the study lacks a comparative analysis of the biomechanics between three Kirschner wire-opening flaps and thyroid dynamic retraction flaps, and further research is needed in the later stage. While a thyroid retractor was utilized to open the skin flap in this study, future considerations may involve designing a new retractor that is more suitable for opening the calcaneal skin flap.

## 5 Conclusions

The “dynamic hook technique” combined with the Allgöwer-Donati suture method demonstrates effectiveness in preventing incision-related complications and reducing postoperative pain after open reduction and internal fixation for calcaneal fractures, thus warranting its promotion in clinical applications.

## Data Availability

The original contributions presented in the study are included in the article/Supplementary Material, further inquiries can be directed to the corresponding authors.
